# Identification of serum biomarkers in dogs naturally infected with *Babesia canis canis* using a proteomic approach

**DOI:** 10.1186/1746-6148-10-111

**Published:** 2014-05-12

**Authors:** Josipa Kuleš, Vladimir Mrljak, Renata Barić Rafaj, Jelena Selanec, Richard Burchmore, Peter D Eckersall

**Affiliations:** 1 Department of Chemistry and Biochemistry, Faculty of Veterinary Medicine, University of Zagreb, Zagreb, Croatia; 2 Clinic for Internal Diseases, Faculty of Veterinary Medicine, University of Zagreb, Zagreb, Croatia; 3 Institute of Infection, Immunity and Inflammation, College of Medical, Veterinary and Life Sciences, University of Glasgow, Glasgow, UK

**Keywords:** Dog, Babesiosis, Acute phase proteins, Serum biomarkers, Proteomics, 2-dimensional electrophoresis

## Abstract

**Background:**

Canine babesiosis is a tick-borne disease that is caused by the haemoprotozoan parasites of the genus *Babesia.* There are limited data on serum proteomics in dogs, and none of the effect of babesiosis on the serum proteome. The aim of this study was to identify the potential serum biomarkers of babesiosis using proteomic techniques in order to increase our understanding about disease pathogenesis.

**Results:**

Serum samples were collected from 25 dogs of various breeds and sex with naturally occurring babesiosis caused by *B. canis canis*. Blood was collected on the day of admission (day 0), and subsequently on the 1st and 6th day of treatment.

Two-dimensional electrophoresis (2DE) of pooled serum samples of dogs with naturally occurring babesiosis (day 0, day 1 and day 6) and healthy dogs were run in triplicate. 2DE image analysis showed 64 differentially expressed spots with p ≤ 0.05 and 49 spots with fold change ≥2. Six selected spots were excised manually and subjected to trypsin digest prior to identification by electrospray ionisation mass spectrometry on an Amazon ion trap tandem mass spectrometry (MS/MS). Mass spectrometry data was processed using Data Analysis software and the automated Matrix Science Mascot Daemon server. Protein identifications were assigned using the Mascot search engine to interrogate protein sequences in the NCBI Genbank database.

A number of differentially expressed serum proteins involved in inflammation mediated acute phase response, complement and coagulation cascades, apolipoproteins and vitamin D metabolism pathway were identified in dogs with babesiosis.

**Conclusions:**

Our findings confirmed two dominant pathogenic mechanisms of babesiosis, haemolysis and acute phase response. These results may provide possible serum biomarker candidates for clinical monitoring of babesiosis and this study could serve as the basis for further proteomic investigations in canine babesiosis.

## Background

Canine babesiosis is a tick-borne disease that is caused by the haemoprotozoan parasites of the genus *Babesia*[1]*. Babesia* is one of the most ubiquitous and widespread blood parasite in the world based on number and distribution of species in animals. It can be compared with the *Plasmodium*, with which it shares phylogenetic proximity and numerous biological features. There are three antigenically different subspecies of *Babesia canis*: *B. canis canis*, *B. canis vogeli* and *B. canis rossi*[2,3]*.* Canine babesiosis caused by *B. canis canis* is a very common cause of morbidity of dogs in Croatia [4-7].

The typical signs of uncomplicated babesiosis are pale mucous membranes, fever, anorexia, depression, splenomegaly and water hammer pulse. The clinical manifestation of the complicated form is variable and includes acute renal failure, cerebral babesiosis, coagulopathy, icterus and hepatopathy, immune-mediated haemolytic anaemia (IMHA), peracute babesiosis, acute respiratory distress syndrome (ARDS), haemoconcentration and shock [8]. Much of the disease process in babesiosis could be explained by the host inflammatory response to the parasite, rather than the parasite itself. Parasitised erythrocytes induce acute phase response and activation of coagulation system. The pathological mechanisms include endothelial cell activation and damage, vascular permeability increase, tissue hipoxia, abnormal perfusion and leukocyte infiltration. These could lead to microcirculation blockade, disseminated intravascular coagulation, and, in severe cases, to systemic inflammatory response syndrome (SIRS) and multiple organ dysfunction syndrome (MODS).

Proteomics has become one of the most significant post-genomic era tools due to advances in proteomic technologies which have allowed an extended experimental approach to investigation of biological systems. The use of proteomic methods in animal sciences can help researchers to identify key proteins and their changed expression in response to different experimental conditions. In a general sense, the research field of host-pathogen interactions is very important due to its impact and importance of infectious diseases that threaten human and animal health. Naturally occurring animal diseases can be studied to improve the health of animals which in turn may lead to improvements in the characterization, clinical management and treatment of human diseases through comparative medicines [9]. There are limited data on serum proteomics in dogs [10], and none on babesiosis.

The aim of this study was to identify potential serum biomarkers using proteomic techniques and to increase our understanding about disease pathogenesis.

## Methods

### Sample collection

Serum samples were collected from 25 dogs of various breeds and sex with naturally occurring babesiosis caused by *B. canis canis*, admitted to Clinic for Internal Diseases, Faculty of Veterinary Medicine, University of Zagreb, Croatia. This study was approved by the Committee on the Ethics of the University of Zagreb, Faculty of Veterinary Medicine (Permit Number: 640-01/06-17/30; 61-01/139-06-03), and informed owner consent was granted in each case. There were 8 females and 17 males, aged from 2 months to 10 years (average age 3 years and 1 month). Eleven dogs were mixed breed, 5 were Labrador Retriever, 2 were Croatian Sheepdog, and one dog of Golden Retriever, American Staffordshire Terrier, German Shepard, Doberman, Alpine Dachsbracke and Alaskan Malamute. Blood was collected on the day of admission (day 0, labeled as B0), and subsequently on the 1st (day 1, B1) and 6th day (day 6, B6) of treatment. The diagnosis of babesiosis was confirmed by demonstration of the parasites within the infected erythrocytes in thin blood smears stained with May-Grünwald-Giemsa stain. Subspecies were confirmed using PCR (polymerase chain reaction). One dose (6 mg/kg) of imidocarb dipropionat (Imizol® 12%, Schering-Plough) was administered to all the dogs subcutaneously on the day of admission. All dogs survived and successfully recovered from disease. Serum was also collected from 10 healthy dogs and they were control group (labeled as C). These dogs were considered healthy based on physical examination and haematological and biochemical data. All dogs were mixed breed, aged from 2 to 10 years, 6 of them were males and 4 females.

### Two-dimensional electrophoresis (2DE) and 2DE analysis

Serum samples were pooled into 4 groups (day 0, day 1, day 6 and control). For making the pool, equal amount of serum was used from each animal. The protein concentrations of the serum samples were measured using the Bradford assay (Bio-Rad Laboratories, Inc., Redmond, WA, USA). Sera from each pool were suspended in rehydration buffer (8 M urea, 2% CHAPS, 0.001% bromophenol blue, 45 mM DTT, 0.2% Bio-lyte pH 3–10) in order to archive final protein concentration of 200 μg/μl. 200 μl of sample was loaded onto a 11 cm IPG strip (immobilized pH gradient, pH 3–10, linear, Biorad Ltd, Hemel Hempstead UK). Isoelectric focusing was performed (Protean IEF Cell, Biorad Ltd, Hemel Hempstead UK) with combined rehydration and focusing step carried out over 17 h with a total of 35,000 V-h. Then the IPG strips were first equilibrated for 10 min in equilibration buffer 1 (6 M urea, 0.375 M Tris–HCl pH 8.8, 20% glycerol, 2% SDS, 2% DTT) and subsequently in equilibration buffer 2 (6 M urea, 0.375 M Tris–HCl pH 8.8, 20% glycerol, 2% SDS, 2.5% iodoacetoamide) for 10 min. Electrophoresis in the second dimension was carried out using Criterion precast gels (XT Bis-Tris Gel, 4-12% polyacrylamide gel, IPG + 1 well, 11 cm IPG strip; Bio-Rad) at 200 V for 45–50 minutes. 2DE gels were then stained with colloidal Coomassie blue G250 (0.12% G250, 20% v/v methanol, 10% v/v o-phosphoric acid, 10% w/v ammonium sulphate) and destained in 5% v/v acetic acid. Molecular mass and pI values were estimated by running a sample containing serum proteins together with a mixture of protein standards (Bio-Rad). Each pool was analyzed in triplicate. The stained gels were scanned (UMAX PowerlookIII, USA) and digitized images of gels were analyzed using the Progenesis Same Spot software (Nonlinear Dynamics Ltd, Newcastle, UK) to identify protein spots which were differentially expressed through time (power >0.8 and ANOVA significance score of <0.05 between experimental groups).

### Trypsin digestion and MS analysis

The selected spots were manually excised from each gel and destained for 30 min using 0.5 ml 100 mM ammonium bicarbonate and then with 50% acetonitrile/100 mM ammonium until gel pieces were transparent. The gels were dehydrated for 10 min with acetonitrile (100%) and dried for 30 min using a Speed-Vac system. To prepare trypsin solution, 25 mM ammonium bicarbonate was added to trypsin (sequencing grade modified Porcine Trypsin, Promega). The trypsin solution (10 μl) was pipetted onto each dried protein spot and incubated overnight at 37°C. To extract the peptide fragments from the tryptic digests, 20 μl of 5% (v/v) formic acid were added and incubated for 20 min. Thereafter, 20 μl of acetonitrile was added to gel pieces and incubated for 20 min. After each step, the supernatants were pooled and dried using a Speed-Vac system.

Peptides were solubilized in 0.5% formic acid and fractionated on a nanoflow ultra high-performance liquid chromatography (uHPLC) system (Thermo RSLCnano) before online analysis by electrospray ionisation (ESI) mass spectrometry on an Amazon ion trap tandem mass spectrometry (MS/MS) (Bruker Daltonics). Peptide separation was performed on a Pepmap C18 reversed phase column (LC Packings), using a 5 - 85% v/v acetonitrile gradient (in 0.5% v/v formic acid) run over 45 min at a flow rate of 0.2 l/min. Mass spectrometry (MS) analysis was performed using a continuous duty cycle of survey MS scan followed by up to ten MS/MS analyses of the most abundant peptides, choosing the most intense multiply charged ions with dynamic exclusion for 120 s.

### Protein identification

MS data was processed using Data Analysis software (Bruker) and the automated Matrix Science Mascot Daemon server (v2.1.06). Protein identifications were assigned using the Mascot search engine to interrogate protein sequences in the NCBI Genbank database, allowing a mass tolerance of 0.4 Da for both MS and MS/MS analyses. All known contaminants (trypsin auto proteolysis and known keratin peaks) were excluded during the process. Search parameters included mammals as taxonomy; trypsin as proteolytic enzyme, a missed cleavages up to 1; fixed modification: carbamidomethyl (C); variable modification: oxidation (M).

### APP assays

CRP and haptoglobin levels were determined using an automated analyser Pentra 400 (Horiba UK Ltd, Northampton, UK) using established methods (ReactivLab, Avacta Animal Health, Wetherby, UK) as described in Gow et al. [11] and Crawford et al. [12]. The canine CRP assay is an immunoturbidimetric assay. Briefly, formed antibody-antigen complex is measured turbidimetrically by the spectrophotometer, and the absorbance is directly proportional to the amount of CRP present in the serum sample [11]. Haptoglobin was assayed with a biochemical assay in which a sample of serum is incubated with haemoglobin in addition of activator to form an Hp-Hb complex [12]. The pH is then reduced by addition of reagent which destroys the peroxidase activity of Hb, except for the Hb molecules bound to Hp. The preservation of the peroxidase activity of the Hb is directly proportional to the amount of Hp present in the sample [12].

### Statistical analysis

Statistical analysis for APP assays were performed using a statistical computer application Statistica 8 (statistical software program Statistica 8 for Windows, StatSoft Inc.). Distribution of data were tested by Kolmogorov-Smirnov test, and as they were not distributed normally, the Friedman test was used for comparing multiple dependent samples, and Mann–Whitney U test to identify statistically significant differences between healthy and diseased dogs. Differences with a P-value <0.05 were considered statistically significant.

## Results

### Proteomic profile of canine babesiosis

**Figure 1:**
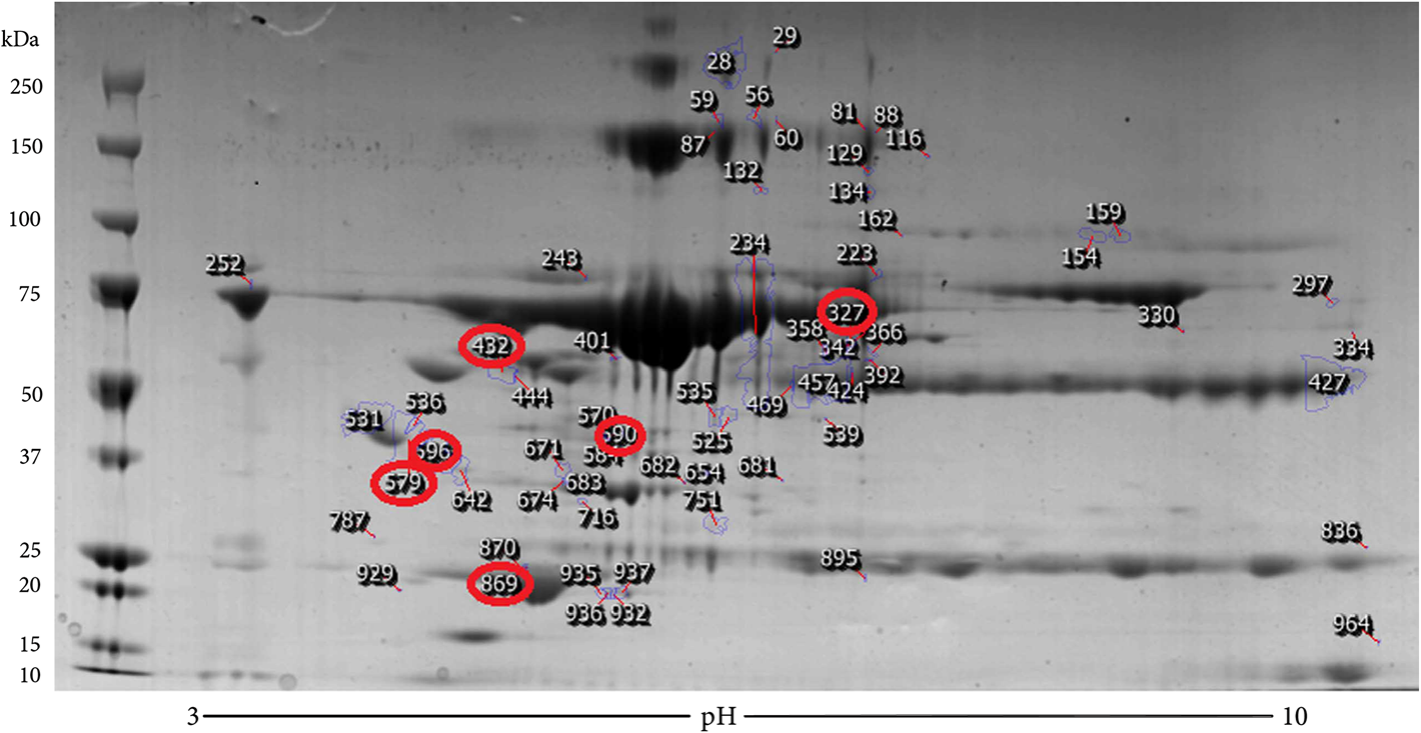
**Reference image 2DE map of differentially expressed spots with marked selected spots.** Representative 2-DE map of canine serum obtained by performing the first dimension (IEF) on IPG strips pH 3–10 and the second dimension on 4–12% gradient SDS-PAGE gels. The protein spots were visualized by Coomassie blue staining. The indicated spots were excised from gel and identified by MS/MS. Mr standard values are indicated on the left.

**Figure 2:**
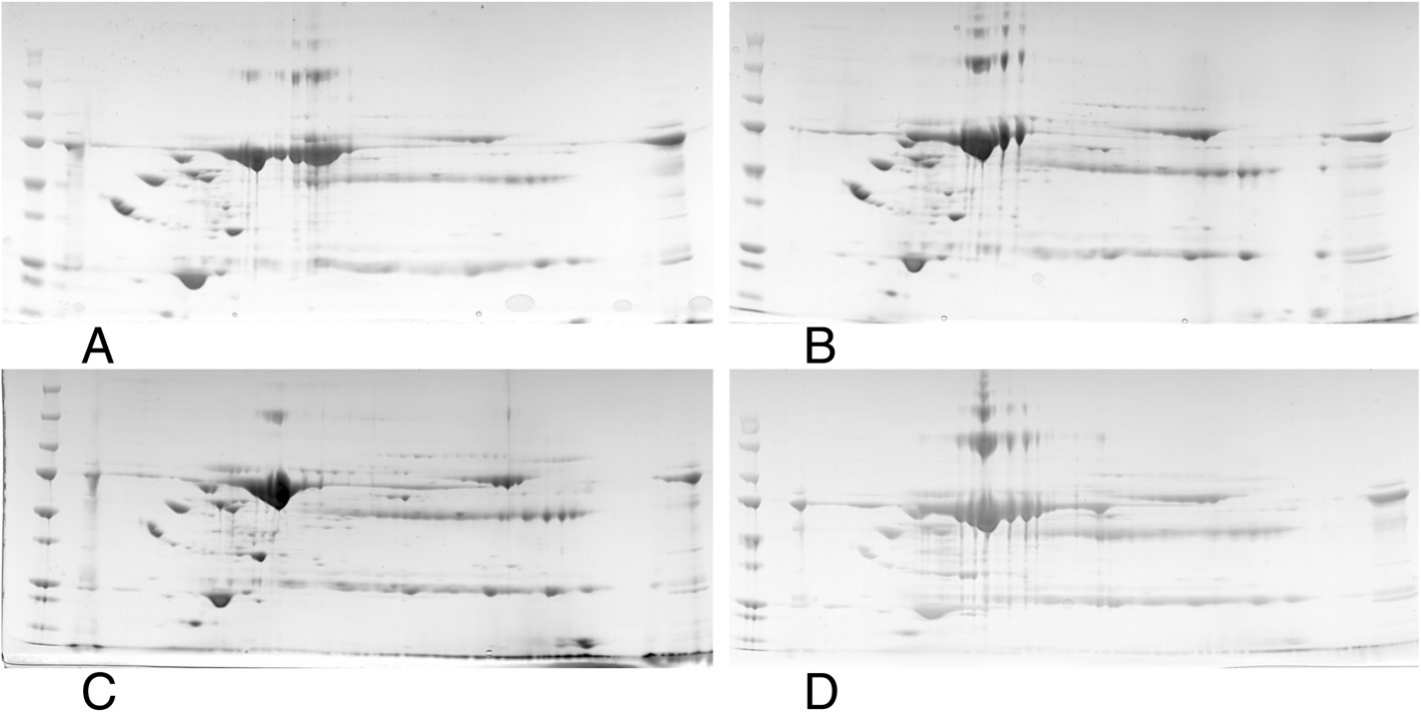
**Representative images of 2-dimensional electrophoresis gels of all experimental groups. A)** dogs with babesiosis before treatment, **B)** dogs with babesiosis on the 1st day after admission, **C)** dogs with babesiosis on the 6th day after admission, **D)** healthy dogs.

2D electrophoresis of pooled serum samples of dogs with naturally occurring babesiosis (day 0, day 1 and day 6) and healthy dogs were run in triplicate. 2D image analysis showed 64 differentially expressed spots with ANOVA P ≤ 0.05 and 49 spots with fold change ≥2. In total, there were 37 spots with P ≤ 0.05 and fold change ≥2, with power >0.8 (Figure 1). A representative gel images for each of the experimental groups are given in the Figure 2. Six spots were selected for running on mass spectrometry (namely, spots 596, 579, 432, 590, 869 and 327). Their fold change and expression level between different experimental groups is given in Table 1. For inclusion in list criteria were Mascot score higher than 100 and sequence coverage over 10%. The summary of identified proteins is given in the Table 2. Identification of proteins for each experimental group (B0, B1, B6, control) are presented as Additional files 1, 2, 3 and 4.

**Table 1:** Protein expression change for each spot between experimental groups

**Spot no.**^**a**^	**P-value (ANOVA)**	**Fold change**	**Log normalised volume**			
			**B0**	**B1**	**B6**	**CONTROL**
327	0.004	8.1	5.11 ± 0.63	4.46 ± 0.22	4.4 ± 0.14	4.29 ± 0.11
869	0.010	3.4	3.24 ± 0.3	3.29 ± 0.24	2.92 ± 0.34	2.76 ± 0.2
590	0.010	3.2	3.52 ± 0.21	3.21 ± 0.25	3.11 ± 0.33	3.03 ± 7.00e-002
579	0.019	4.4	5.73 ± 0.4	6.03 ± 0.25	5.59 ± 0.34	5.37 ± 0.33
596	0.019	3.6	5.57 ± 8.59e-002	5.91 ± 0.49	5.43 ± 0.28	5.4 ± 7.71e-002
432	0.020	2.0	4.81 ± 0.11	5.06 ± 0.11	4.92 ± 0.19	5.07 ± 0.19

^a^Number refer to protein spots indicated in Figure 1.

**Table 2:** Protein identification of differentially expressed proteins

**Protein name**	**Accession number**^**a**^	**Number of unique peptides**^**b**^	**Sequence coverage (%)**^**b**^	**Mascot score**^**b**^	**Biological function**	**Spot no.**^**c**^
Alpha-1-acid glycoprotein	gi|345777714	25	53	628	Acute phase response	596, 579
Apolipoprotein A-I	gi|73955106	70	72	1834	Cholesterol, lipid and steroid metabolism	869, 432
					Lipid transport	
Antithrombin-III	gi|359320010	5	19	161	Blood coagulation	432
Vitamin D-binding protein	gi|73975215	24	57	543	Vitamin D metabolic process	432
Apolipoprotein A-IV	gi|345799905	66	72	1401	Removal of superoxide radicals	590
					Lipid metabolism	
Complement C3	gi|359322249	16	15	356	Complement activation (classical and alternative pathway)	590
					Inflammatory response	
Serotransferrin	gi|73990142	25	40	672	Cellular iron ion homeostasis	327
					Iron ion transport	
Hemopexin	gi|73988725	12	44	273	Cellular iron ion homeostasis	327
Alpha-2-HS-glycoprotein	gi|359323766	7	40	273	Acute phase response	596, 432
Haptoglobin	gi|123511	5	41	159	Cellular iron ion homeostasis	579
					Acute phase response	
Alpha-2-antiplasmin	gi|345805038	3	13	101	Acute phase response	327
					Fibrinolysis	
Clusterin	gi|50979240	31	25	864	Apoptosis	596, 579
					Complement pathway	
Leucine-rich-α2-glycoprotein	gi|73987375	24	50	625	Acute phase response	432
Zinc-alpha-2-glycoprotein	gi|73958037	15	46	310	Immune response	579
Immunoglobulin gamma heavy chain B	gi|17066526	12	27	206	Immune response	432
					Complement activation	
Immunoglobulin gamma heavy chain C	gi|17066528	10	20	222	Immune response	432
IgA heavy chain constant region	gi|598107	7	22	124	Immune response	327

^a^Accesion number from NCBI Genbank database for *Canis lupus familiaris.*

^b^Data are representatives of four separate experimental group.

^c^Number refer to protein spots indicated in Figure 1.

### APPs in canine babesiosis

**Figure 3:**
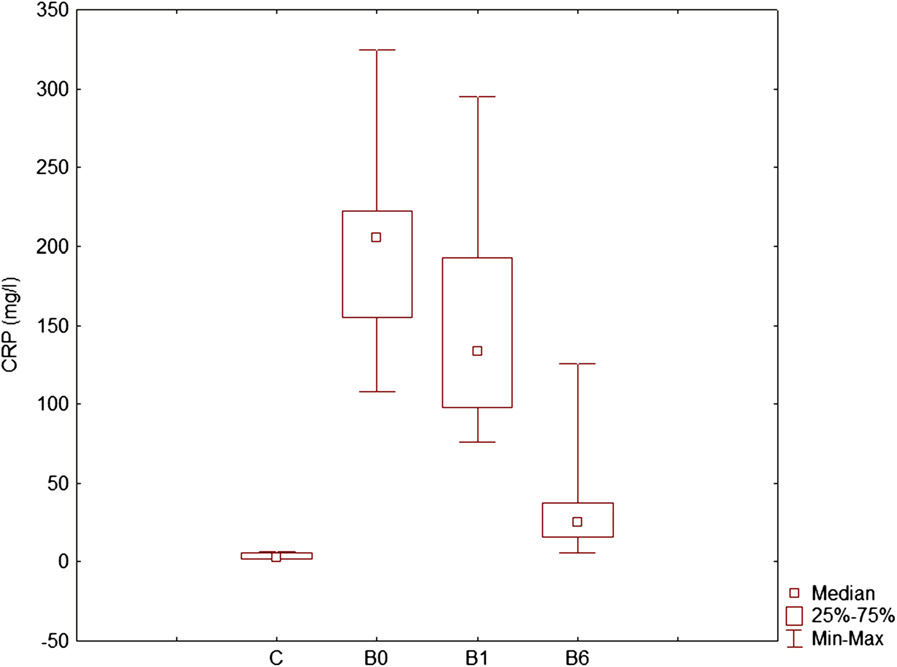
Concentration of CRP in healthy (control) dogs (C), dogs with babesiosis before treatment (B0), on the 1st day (B1) and 6th day (B6) after admission.

**Figure 4:**
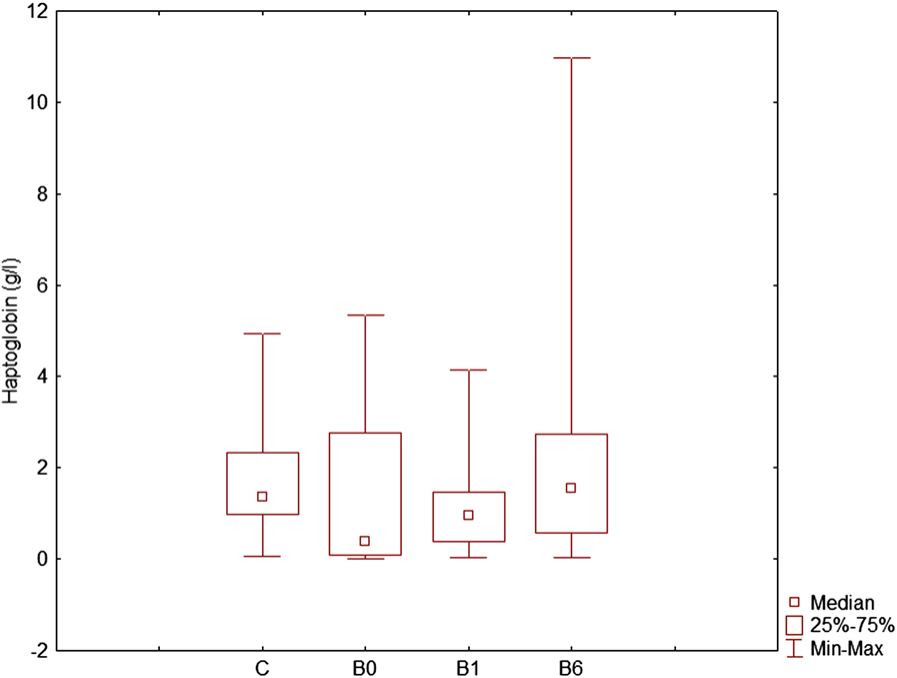
Concentration of haptoglobin in healthy (control) dogs (C), dogs with babesiosis before treatment (B0), on the 1st day (B1) and 6th day (B6) after admission.

Levels of CRP and haptoglobin were determined on day 0, day 1 and day 6 in serum of dogs with babesiosis and healthy dogs (Figures 3 and 4). Non-parametric Friedman test was used to identify statistically significant differences. CRP was significantly different between each group of infected dogs, as well as compared with healthy dogs, while haptoglobin showed significant decrease on day 1 of disease comparing to day 6 and to healthy dogs.

## Discussion

Proteomics is a rapidly maturing essential tool in the “omics” age following the huge success of genomics study. Studying proteins has been subject of interest for many researchers as any cell responses to stimuli or stress are manifested by the alteration of protein expression level. In this study, we used a proteomic approach to analyze the alterations in canine serum proteome due to *B. canis canis* infection. 2DE gels on three replicates generated from the infected serum samples were compared with the gels from healthy serum samples, followed by MS/MS analysis, which revealed several significantly differential serum proteins. Analytical problems in proteomics are related to the diversity of protein expression (multiple protein forms) and the dynamic expression range (protein abundance). In the majority of our selected spots, multiple proteins were identified per spot due to similar migration patterns and insufficient resolving power. Proteins can migrate to multiple spots on 2D gels for a variety of reasons, including differential protein processing and posttranslational or artefactual modifications. The protein products from multiple genes also can run to the same coordinates on a gel. Both differential migration and comigration of proteins complicate comparative, quantitative pattern analyses of 2D gels [13].

A number of differentially expressed serum proteins involved in inflammation mediated acute phase response, complement and coagulation cascades, apolipoproteins and vitamin D metabolism pathway were identified in dogs with babesiosis. Some proteins in the particular spot were present in all groups, while others are only in some of them. All spots, except spot 432, were up-regulated in diseased dogs while the down-regulation of spot 432 resulted from decreased levels of antithrombin III, vitamin D binding protein and albumin in dogs with babesiosis comparing to healthy dogs.

In order to access inflammatory status of dogs and extent of acute phase response, we measured CRP in serum of dogs. Elevated CRP levels have been reported in dogs after surgical trauma [14], during infections including leishmaniasis [15], ehrlichiosis [16], babesiosis [5], leptospirosis [17], bordetellosis [18], parvovirus infection [17] and pyometra [19], and with noninfectious conditions including neoplasia [20], autoimmune disorders [20], acute pancreatitis [21], inflammatory bowel disease [22] and cardiac valvular disease [23]. Several researchers have explored the acute phase response and specifically CRP in canine babesiosis [5]. In our study in dogs infected with *B. canis canis* CRP concentrations were significantly higher than in healthy dogs and CRP levels decreased with time and treatment. CRP is a biomarker and indicator of inflammation, and plays a role of an acute phase reactant in babesiosis.

Using proteomic approach, we have found increased expression level of several further acute phase proteins including alpha-1-acid glycoprotein, leucine-rich-alpha-2-glycoprotein, hemopexin, serotransferrin and alpha-2-HS-glycoprotein, in dogs with babesiosis suggesting a possible association with the disease pathogenesis.

Alpha-1-acid glycoprotein (AGP) is a positive acute phase reactant in all mammals investigated to date, except for the pig, with antiinflammatory and immunomodulating activities [24]. Lobetti et al., [25] found increased levels of AGP in dogs invaded with *B. rossi*.

The leucine-rich repeat family of proteins, including leucine-rich-alpha-2-glycoprotein (LRG), have been shown to be involved in protein-protein interaction, signal transduction, and cell adhesion and development [26]. LRG was recently identified as a potential disease activity marker in Crohn’s disease and rheumatoid arthritis and a novel serum biomarker for monitoring disease activity during therapy in autoimmune patients [27,28]. It was hypothesized that serum LRG would potentially surrogate for CRP.

*α*2-Heremans–Schmid-(*α*2-HS) glycoprotein (A2HSG) is a plasma protein synthesized in liver and enriched in bone, a homologue of bovine fetuin-A. This protein is a negative acute-phase protein whereas its level declines following infection, inflammation and malignancy. It plays a role in augmentation of phagocytosis of neutrophils by macrophages, thus acting as an anti-inflammatory molecule [29].

Three APP involved in haemoglobin and iron metabolism and transport, haptoglobin, hemopexin and serotransferrin, indicate the role of haemolysis in the course of babesiosis.

Hemopexin, another up-regulated protein in our study, provides the second line of defense preventing haemoglobin-mediated oxidative damage during the intravascular haemolysis and heme-bound iron loss [30]. Its synthesis is induced under inflammatory conditions during disease progression, resulting in haemoglobin degradation and reactive oxygen species (ROS) generation in parasitized erythrocytes. While hemopexin helps in scavenging free heme, the free iron is taken up and transported by serotransferrin [31]. Haptoglobin was identified only in dogs with babesiosis on 6th day after treatment. We determined levels of haptoglobin in serum of all dogs, in order to achieve better understanding of haemolysis in babesiosis. In our study, we found significant decrease of haptoglobin levels on day 1 of disease comparing to day 6 and to healthy dogs. Some authors found increase and some decrease of haptoglobin during babesiosis [5,32] suggesting that serum concentration of Hp can be influenced by other factors than acute phase response. In this context, it is important to note that Hp binds to free haemoglobin, and the complex is cleared by the reticuloendothelial system, resulting in a decrease in serum Hp concentrations. The release of free haemoglobin into the vascular system following a haemolytic crisis or the onset of haemolytic anaemia will therefore cause a marked loss of haptoglobin. Therefore levels of Hp in dogs with *B. canis canis* infection might be the result of an imbalance between the synthesis and the clearance of this protein from the circulation, and in particular may relate to the magnitude of haemolysis. Increase or decrease of Hp levels depends whether inflammatory or haemolytic component dominates. The ability rapidly and accurately to measure Hp levels may be a valuable diagnostic aid, especially in detecting the magnitude of haemolysis in babesiosis.

Lipid metabolism is of great importance in babesiosis because of the catabolic reaction within the acute phase response and because of the fact that the majority of haemoparasites (including *Babesia*) cannot synthesize their own lipids, so they take them up from host plasma (predominantly high density lipoproteins (HDL)) [33].

The main protein component of HDL, apolipoprotein A-I (apoA-I) participates in the reverse transport of cholesterol from tissues to the liver for excretion by promoting cholesterol efflux from tissues and by acting as a cofactor for the lecithin cholesterol acyltransferase (LCAT). ApoA-1 is known to have anti-inflammatory effects, and thus apoA-1 increase (spot 869) may contribute to the limitation of inflammatory processes. In humans, this protein belongs to negative APP and lowered plasma levels of apoA-I is considered to be correlated to an increased risk of cardiovascular diseases and atherosclerosis. The distribution of plasma lipids and lipoproteins in dogs is quite unlike that in humans, with dogs having approximately five to six times as much HDL as LDL [34].

Apolipoprotein A-IV (apo A-IV) (spot 590) is a 46 kDa protein that circulates freely in blood or associates with chylomicrons and high-density lipoprotein. Apo A-IV has a role in radicals catabolism, activation of lipoprotein lipase by apoC-II and activation of LCAT. It has been proposed that Apo A-IV plays a protective role against atherosclerosis and reverses cholesterol transport [35,36]. Because of oxidative damage caused by free radicals generated during the course of babesiosis [37], increased levels of apo A-IV may have antioxidative activity as an oxidation inhibitor of lipids and lipoproteins.

A primary consequence of *Babesia* infections are haemostasis disturbances together with inflammation-related complications. It has been well documented that interplay between inflammation and coagulation pathways lead to activation of both systems [38]. Thrombin generated as a by-product of the coagulation process can further promote inflammatory responses [39], while inflammation suppresses anticoagulation mechanisms [40].

Antithrombin-III (AT III) is a major endogenous anticoagulant and antiinflammatory agent. It is a circulating glycoprotein that can inhibit thrombin directly and via a number of upstream factors. The AT III levels were significantly decreased in dogs with babesiosis (spot 432) indicating an imbalance in haemostasis defined by the overproduction of thrombin and fibrin [41]. This could be a consequence of inhibitor breakdown and/or liver dysfunction affecting inhibitor production in dogs with babesiosis. Low AT III levels are likely to be due to increased consumption from thrombin binding in thrombin–antithrombin (TAT) complexes.

Alpha-2-antiplasmin is a major inhibitor and regulator of fibrinolysis and one of the essential factors involved in haemostasis. It is a member of the serine proteinase inhibitor (serpin) family and inhibits proteases in general, including trypsin, chymotrypsin, plasma kallikrein, but its main physiological activity is very rapid inhibition of plasmin by forming a stable complex with this proteinase [42]. Thus, the level of alpha-2-antiplasmin in circulation may be a crucial determinant of fibrinolysis in babesiosis.

Complement activation in babesiosis was confirmed by clusterin and C3 increased expression. Complement component 3 (C3) plays a central role in the activation of complement system. Its activation is required for both classical and alternative complement activation pathways. Clusterin (CLU, complement lysis inhibitor, apolipoprotein J) is a heterodimeric glycoprotein produced by a wide array of tissues and found in most biologic fluids. It participates in many different biological reactions including regulation of the terminal complement cascade, lipid transport, initiation of apoptosis, endocrine secretion, promotion of cell interactions, tissue remodeling, adhesion, membrane protection [43]. Recently, upregulated expression of CLU has been reported in tumor pathogenesis and progression, Alzheimer’s disease, atherosclerosis, renal diseases [44,45].

Vitamin D binding protein, also known as group-specific component globulin (Gc-globulin), belongs to the albumin superfamily of binding proteins. One well-recognized function of vitamin D binding protein is to act as a carrier protein for vitamin D and its plasma metabolites. Clinical studies and animal models have also shown its role as an actin-scavenging protein in the vascular and extracellular system [46]. To date, vitamin D binding protein has been recognized widely as a protein with markedly decreased concentrations in inflammatory and necrotic diseases. The extent of the decrease may have prognostic significance for patient outcomes. Vitamin D binding protein has other potential roles in responses to acute tissue injury through conversion to a macrophage-activating factor, neutrophil chemotactic activity, and enhancement of C5a-mediated signaling. Low vitamin D binding protein concentrations have been demonstrated to be prognostic markers in situations of severe organ damage, such as fulminant hepatic failure, acetaminophen (paracetamol) overdose, multiple trauma, and multiple organ failure [47-50]. Several studies have shown that low vitamin D binding protein concentrations are associated with a poor prognosis for survival and an increased risk of developing MODS in sepsis [51]. Decreased vitamin D binding protein levels in babesiosis can be consequence of consumption due to haemolysis and increased actin removal.

Our findings confirmed two dominant pathogenic mechanisms of babesiosis, haemolysis and acute phase response [52,53]. The role of haemolysis in the course of babesiosis was demonstrated by haptoglobin, hemopexin and serotransferrin expression changes. Tissue hypoxia, which is a common feature in babesiosis, is probably one of the major causes for the release of cytokines, oxygen free radicals, nitric oxide and other inflammatory mediators. As a consequence, acute phase response was triggered and demonstrated throughout wide variety of acute phase proteins (alpha-1-acid glycoprotein, leucine-rich-alpha-2-glycoprotein, hemopexin, serotransferrin, alpha-2-HS-glycoprotein, albumin). Also, release of ROS and consequent oxidative damage, lead to increased expression of apolipoproteins (apoA-I and apoA-IV) with antioxidative activity. Impairment of coagulation and fibrinolytic system was demonstrated throughout consumption of AT III due to the coagulation activation. And finally, vitamin D binding protein, as a novel biomarker for MODS in different conditions, can be a possible target for further validation in babesiosis.

## Conclusions

The search for new biomarkers for early detection, monitoring and prognosis of canine babesiosis is an active area of interest. The objective in utilizing proteomic techniques of this study was to identify differentially expressed protein biomarkers in babesiosis. As a result, a number of differentially expressed serum proteins involved in inflammation mediated acute phase response (alpha-1-acid glycoprotein, leucine-rich-alpha-2-glycoprotein, haptoglobin, hemopexin, serotransferrin, alpha-2-HS-glycoprotein, albumin), complement and coagulation cascades (AT III, CLU, C3, alpha-2-antiplasmin), apolipoproteins (apoA-I and apoA-IV) and vitamin D metabolism pathway were identified in dogs with babesiosis. These results may provide possible serum biomarker candidates for clinical monitoring of babesiosis. The major limitation of this study is insensitivity of 2-DE gel methods used for proteome analysis. Thus, the depletion of major proteins has been suggested to be a potential strategy for enhancing detection sensitivity in serum. However, this study could serve as the basis for further proteomic investigations in canine babesiosis.

## Abbreviations

CRP: C-reactive protein; 2DE: Two-dimensional electrophoresis; MS: Mass spectrometry; MS/MS: Tandem mass spectrometry; IMHA: Immune-mediated haemolytic anaemia; ARDS: Acute respiratory distress syndrome; SIRS: Systemic inflammatory response syndrome; MODS: Multiple organ dysfunction syndrome; APP: Acute phase proteins; APR: Acute phase response; Hp: Haptoglobin; PCR: Polymerase chain reaction; IPG: Immobilized pH gradient; ESI: Electrospray ionisation; UHPLC: Ultra high-performance liquid chromatography; AGP: Alpha-1-acid glycoprotein; LRG: Leucine-rich-alpha-2-glycoprotein; ROS: Reactive oxygen species; A2HSG: *α*2-HS- glycoprotein; HDL: High density lipoprotein; ApoA-I: Apolipoprotein A-I; LCAT: Lecithin cholesterol acyltransferase; LDL: Low density lipoprotein; Apo A-IV: Apolipoprotein A-IV; AT III: Antithrombin-III; TAT: Thrombin–antithrombin complex; C3: Complement component 3; CLU: Clusterin.

## Competing interests

PDE is a shareholder and consultant to ReactivLab Ltd, a veterinary diagnostic company with acute phase protein interests. No other author has a financial or personal relationship with other people or organisations that could inappropriately influence or bias the content of the paper.

## Authors’ contributions

JK participated in the 2D gel analyses, laboratory assays, trypsin digestion and drafted the manuscript. JS carried out the sample collection and participated in the statistical analysis. RBR participated in data collection, critical reading and in revising of the manuscript. RB participated in the MS analyses and in revising of the manuscript. VM participated in design of study and coordination and helped to draft the manuscript. PDE designed the study and participated in writing and revising the manuscript. All authors read, commented on, and approved the final manuscript.

## Supplementary Material

Additional file 1 **List of proteins identified in serum of dogs with *B. canis canis *** **at the day of admission.** a) Number refer to protein spots indicated in Figure 1b) Accesion number from NCBI Genbank database for *Canis lupus familiaris.* Click here for file

Additional file 2 **List of proteins identified in serum of dogs with *B. canis canis *** **on the first day.** a) Number refer to protein spots indicated in Figure 1b) Accesion number from NCBI Genbank database for *Canis lupus familiaris.* Click here for file

Additional file 3 **List of proteins identified in serum of dogs with *B. canis canis *** **on the 6th day.** a) Number refer to protein spots indicated in Figure 1b) Accesion number from NCBI Genbank database for *Canis lupus familiaris.* Click here for file

Additional file 4 **List of proteins identified in serum of healthy dogs.** a) Number refer to protein spots indicated in Figure 1b) Accesion number from NCBI Genbank database for *Canis lupus familiaris.* Click here for file
