# Supplementary material for: Identification of serum biomarkers in dogs naturally infected with Babesia canis canis using a proteomic approach
Source: BMC Vet Res. 2014 May 12;10:111. doi: 10.1186/1746-6148-10-111 (PMC4045879; doi:10.1186/1746-6148-10-111)
Supplement: Additional file 1 — List of proteins identified in serum of dogs with B. canis canis at the day of admission. a) Number refer to protein spots indicated in Figure 1b) Accesion number from NCBI Genbank database for Canis lupus familiaris. [file 1746-6148-10-111-S1.docx]

## Additional file 1 - List of proteins identified in serum of dogs with *B. canis* at the day of admission.

| **Spot No.^a^** | **Protein name** | **Accession number^b^** | **Theoretical Mr (kDa)/pI** | **Number of unique peptides** | **Sequence coverage (%)** | **Mascot score** |
| --- | --- | --- | --- | --- | --- | --- |
| 596 | Alpha-1-acid glycoprotein-like  Clusterin precursor  Alpha-2-HS-glycoprotein isoform 1 | gi\|345777714  gi\|50979240  gi\|359323766 | 24/5.4  52/5.6  41/5.7 | 25  11  5 | 53  15  29 | 628  364  160 |
| 579 | Clusterin precursor  Albumin  Alpha-1-acid glycoprotein-like | gi\|50979240  gi\|1351907  gi\|345777714 | 52/5.6  71/5.8  24/5.4 | 13  11  7 | 19  21  39 | 408  235  178 |
| 432 | Leucine-rich alpha-2-glycoprotein  Albumin  Antithrombin-III isoform 1 | gi\|73987375  gi\|3319897  gi\|359320010 | 38/6.2  68/5.4  53/6.3 | 25  14  5 | 40  25  19 | 586  258  161 |
| 590 | Apolipoprotein A-IV  Serum albumin precursor | gi\|345799905  gi\|55742764 | 46/5.6  71/5.5 | 66  42 | 72  62 | 1401  836 |
| 869 | Apolipoprotein A-I | gi\|73955106 | 30/5.3 | 36 | 77 | 1575 |
| 327 | Serotransferrin isoform 1  Albumin  IgA heavy chain constant region  Hemopexin | gi\|73990142  gi\|3319897  gi\|598107  gi\|73988725 | 80/7.7  68/5.4  36/6.1  52/6.9 | 21  15  7  7 | 36  32  35  35 | 560  270  176  133 |

## Number refer to protein spots indicated in Figure 1.

1. Accesion number from NCBI Genbank database for *Canis lupus familiaris*
