# Supplementary material for: Identification of serum biomarkers in dogs naturally infected with Babesia canis canis using a proteomic approach
Source: BMC Vet Res. 2014 May 12;10:111. doi: 10.1186/1746-6148-10-111 (PMC4045879; doi:10.1186/1746-6148-10-111)
Supplement: Additional file 2 — List of proteins identified in serum of dogs with B. canis canis on the first day. a) Number refer to protein spots indicated in Figure 1b) Accesion number from NCBI Genbank database for Canis lupus familiaris. [file 1746-6148-10-111-S2.docx]

## Additional file 2 - List of proteins identified in serum of dogs with *B. canis* on the first day.

| **Spot No.^a^** | **Protein name** | **Accession number^b^** | **Theoretical Mr (kDa)/pI** | **Number of unique peptides** | **Sequence coverage (%)** | **Mascot score** |
| --- | --- | --- | --- | --- | --- | --- |
| 596 | Alpha-1-acid glycoprotein-like  Clusterin precursor  Alpha-2-HS-glycoprotein isoform 1 | gi\|345777714  gi\|50979240  gi\|359323766 | 24/5.4  52/5.6  41/5.7 | 18  6  3 | 53  11  15 | 372  218  114 |
| 579 | Clusterin precursor | gi\|50979240 | 52/5.6 | 19 | 19 | 562 |
| 432 | Leucine-rich alpha-2-glycoprotein  Albumin | gi\|73987375  gi\|3319897 | 38/6.2  68/5.4 | 24  8 | 50  26 | 625  197 |
| 590 | Apolipoprotein A-IV  Albumin | gi\|345799905  gi\|3319897 | 46/5.6  68/5.4 | 66  18 | 71  33 | 1301  470 |
| 869 | Apolipoprotein A-I | gi\|73955106 | 30/5.3 | 70 | 72 | 1834 |
| 327 | Serotransferrin isoform 1  Hemopexin  IgA heavy chain constant region | gi\|73990142  gi\|73988725  gi\|598107 | 80/7.7  52/6.9  38/6.1 | 22  12  7 | 42  44  22 | 593  273  124 |

## Number refer to protein spots indicated in Figure 1.

1. Accesion number from NCBI Genbank database for *Canis lupus familiaris*
