# Supplementary material for: Identification of serum biomarkers in dogs naturally infected with Babesia canis canis using a proteomic approach
Source: BMC Vet Res. 2014 May 12;10:111. doi: 10.1186/1746-6148-10-111 (PMC4045879; doi:10.1186/1746-6148-10-111)
Supplement: Additional file 3 — List of proteins identified in serum of dogs with B. canis canis on the 6th day. a) Number refer to protein spots indicated in Figure 1b) Accesion number from NCBI Genbank database for Canis lupus familiaris. [file 1746-6148-10-111-S3.docx]

## Additional file 3 - List of proteins identified in serum of dogs with *B. canis* on the 6^th^ day.

| **Spot No.^a^** | **Protein name** | **Accession number^b^** | **Theoretical Mr (kDa)/pI** | **Number of unique peptides** | **Sequence coverage (%)** | **Mascot score** |
| --- | --- | --- | --- | --- | --- | --- |
| 596 | Clusterin precursor  Albumin | gi\|50979240  gi\|3319897 | 52/5.6  41/5.7 | 31  4 | 25  24 | 864  107 |
| 579 | Zinc-alpha-2-glycoprotein  Clusterin precursor  Haptoglobin | gi\|73958037  gi\|50979240  gi\|123511 | 36/4.8  52/5.6  37/5.7 | 15  8  5 | 46  25  41 | 310  200  159 |
| 432 | Vitamin D-binding protein isoform 2  Albumin  Alpha-2-HS-glycoprotein isoform 1  Apolipoprotein A-I  Antithrombin-III isoform 1  Immunoglobulin gamma heavy chain C  Immunoglobulin gamma heavy chain B | gi\|73975215  gi\|3319897  gi\|359323766  gi\|73955106  gi\|359320010  gi\|17066528  gi\|17066526 | 55/5.2  71/5.5  41/5.7  30/5.3  52/6.9  53/6.2  53/8.5 | 24  15  7  9  7  10  12 | 57  32  40  58  22  20  27 | 543  289  273  270  232  222  206 |
| 590 | Apolipoprotein A-IV  Serum albumin precursor | gi\|345799905  gi\|55742764 | 46/5.6  71/5.5 | 58  40 | 72  59 | 1117  983 |
| 869 | Apolipoprotein A-I | gi\|73955106 | 30/5.3 | 43 | 77 | 1077 |
| 327 | Serotransferrin isoform 1  Complement C3  Hemopexin  Albumin  Apolipoprotein A-I  Alpha-2-antiplasmin isoform 1 | gi\|73990142  gi\|359322249  gi\|73988725  gi\|3319897  gi\|3915607  gi\|345805038 | 80/7.7  175/6.9  52/6.9  68/5.4  30/5.2  55/6.7 | 25  16  16  9  4  3 | 40  15  44  27  22  13 | 672  356  260  214  161  101 |

## Number refer to protein spots indicated in Figure 1.

1. Accesion number from NCBI Genbank database for *Canis lupus familiaris*
