# Supplementary material for: Identification of serum biomarkers in dogs naturally infected with Babesia canis canis using a proteomic approach
Source: BMC Vet Res. 2014 May 12;10:111. doi: 10.1186/1746-6148-10-111 (PMC4045879; doi:10.1186/1746-6148-10-111)
Supplement: Additional file 4 — List of proteins identified in serum of healthy dogs. a) Number refer to protein spots indicated in Figure 1b) Accesion number from NCBI Genbank database for Canis lupus familiaris. [file 1746-6148-10-111-S4.docx]

## Additional file 4 - List of proteins identified in serum of healthy dogs.

| **Spot No.^a^** | **Protein name** | **Accession number^b^** | **Theoretical Mr (kDa)/pI** | **Number of unique peptides** | **Sequence coverage (%)** | **Mascot score** |
| --- | --- | --- | --- | --- | --- | --- |
| 596 | Alpha-1-acid glycoprotein-like | gi\|345777714 | 24/5.4 | 25 | 48 | 580 |
| 579 | Clusterin precursor  Albumin | gi\|50979240  gi\|229552 | 52/5.6  68/5.8 | 12  9 | 16  17 | 369  218 |
| 432 | Serum albumin precursor  Vitamin D-binding protein isoform 2  Leucine-rich alpha-2-glycoprotein  Apolipoprotein A-I | gi\|55742764  gi\|73975215  gi\|73987375  gi\|73955106 | 71/5.5  55/5.2  38/6.2  30/5.3 | 16  13  11  9 | 37  45  37  48 | 385  332  269  264 |
| 590 | Serum albumin precursor  Apolipoprotein A-IV | gi\|55742764  gi\|345799905 | 71/5.5  46/5.6 | 43  45 | 58  72 | 935  903 |
| 869 | Apolipoprotein A-I  Albumin | gi\|73955106  gi\|3319897 | 30/5.3  68/5.4 | 57  6 | 77  11 | 1492  116 |
| 327 | Serotransferrin isoform 1  Complement C3  Hemopexin  Albumin | gi\|73990142  gi\|359322249  gi\|73988725  gi\|3319897 | 80/7.7  175/6.9  52/6.9  68/5.4 | 10  11  12  4 | 25  12  39  12 | 248  226  215  128 |

## Number refer to protein spots indicated in Figure 1.

1. Accesion number from NCBI Genbank database for *Canis lupus familiaris*
